# Rheumatoid arthritis and hypothyroidism: a bidirectional Mendelian randomization study

**DOI:** 10.3389/fimmu.2023.1146261

**Published:** 2023-08-02

**Authors:** Lincheng Duan, Dongqing Chen, Yue Shi, Senlin Ye, Shulin Dou, Yue Feng

**Affiliations:** ^1^ Chengdu University of Traditional Chinese Medicine, Chengdu, China; ^2^ Meishan Hospital of Traditional Chinese Medicine, Affiliated Meishan Hospital of Chengdu University of Traditional Chinese Medicine, Meishan, China

**Keywords:** rheumatoid arthritis, hypothyroidism, Mendelian randomization, single-nucleotide polymorphisms, genome-wide association study

## Abstract

**Object:**

Though significant correlations between rheumatoid arthritis (RA) and hypothyroidism have been found in earlier observational studies, their underlying causal relationship is still unknown. Mendelian randomization (MR) was used in the current study to assess the bidirectional causation between RA and hypothyroidism.

**Method:**

We gathered summary data from genome-wide association studies (GWASs) of RA and hypothyroidism in people of European descent. Then, using data from the FinnGen consortium, we replicated our findings. Three approaches were employed to assess the causal link between RA and hypothyroidism: MR-Egger, weighted median (WM), and inverse variance weighted (IVW). The pleiotropy and heterogeneity were examined using a variety of techniques, including the MR-Egger intercept, the MR-PRESSO approach, the leave-one-out method, and the Cochran’s Q test.

**Results:**

The study looked at a bidirectional incidental relationship between RA and hypothyroidism. The risk of hypothyroidism increased with RA (IVW odds ratio (OR) = 1.28, 95% confidence interval (CI) = 1.18–1.39, *P* = 8.30E-10), as did the risk of secondary hypothyroidism (IVW OR = 1.12, 95% CI = 1.05–1.21, *P* = 9.64E-4). The results of reverse MR analysis revealed that hypothyroidism (IVW OR = 1.68, 95% CI = 1.51–1.88, *P* = 4.87E-21) and secondary hypothyroidism (IVW OR = 1.74, 95% CI = 1.50–2.01, *P* = 1.91E-13) were linked to an increased risk of RA. Additionally, we obtain the same results in the duplicated datasets as well, which makes our results even more reliable. This study revealed no evidence of horizontal pleiotropy.

**Conclusion:**

The present study established a bidirectional causal link between RA and hypothyroidism. However, it differs slightly from the findings of prior observational studies, suggesting that future research should concentrate on the interaction mechanisms between RA and hypothyroidism.

## Introduction

Chronic and systemic autoimmune illness rheumatoid arthritis (RA) can cause ongoing inflammatory polyarthritis and joint damage, increases the likelihood of deformity or disability. A recent study determined that over the past 40 years, the prevalence of RA was nearly 0.46 percent worldwide and is expected to increase in the future (1). In addition, studies have found that RA may be linked to a number of autoimmune conditions, such as hypothyroidism. A frequent side effect of autoimmune rheumatic disease (ARD) is hypothyroidism. The main manifestations are musculoskeletal discomfort, fatigue, edema, and dryness. Hypothyroidism and rheumatoid arthritis have been linked in previous research.

RA and hypothyroidism are the most prevalent chronic specific autoimmune diseases. The etiology of these two diseases remains to be clarified, but they mainly involve genetic, environmental, and other factors, and the genes that lead to the pathogenesis of these two diseases still overlap. But that’s not enough to explain the relationship. Previous research found that RA patients frequently have thyroid dysfunction, with a prevalence of 6% to 34% (2). Autoimmune diseases usually have similar pathological pathways, which means that there may be an association between RA and hypothyroidism (3). A recent study confirmed a significant link between rheumatoid arthritis and hypothyroidism (4). But some studies found no correlation (5). Although the association between RA and hypothyroidism has been actively studied for many years, there is still a lack of clear data to support this view. We speculate that RA and hypothyroidism may have similar pathogenesis and exist a relationship, so it is particularly necessary to determine the causal association between RA and hypothyroidism.

Limitations of traditional observational studies include confounding variables and reverse causality. Due to possible biases in confounding factors, these previous observational data may be limited in correlation inference, and the conclusions may even be disputed. A randomized controlled trial (RCT) may determine the causal connection between RA and hypothyroidism, but it consumes a lot of money and time. Therefore, the Mendelian randomization (MR) method is used specifically for demonstrating whether the link between risk variables and outcomes can be explained by causal effects (6). MR study used single nucleotide polymorphisms (SNPs) as instrumental variables (IVs) to examine the causal relationship between exposure and outcome. It avoided confounding and reverse causality because genetic variants were identified at the time of conception. In the current study, we used a two-sample bidirectional MR analysis to determine if RA and hypothyroidism are causally related.

## Materials and methods

### Study design

**Figure 1:**
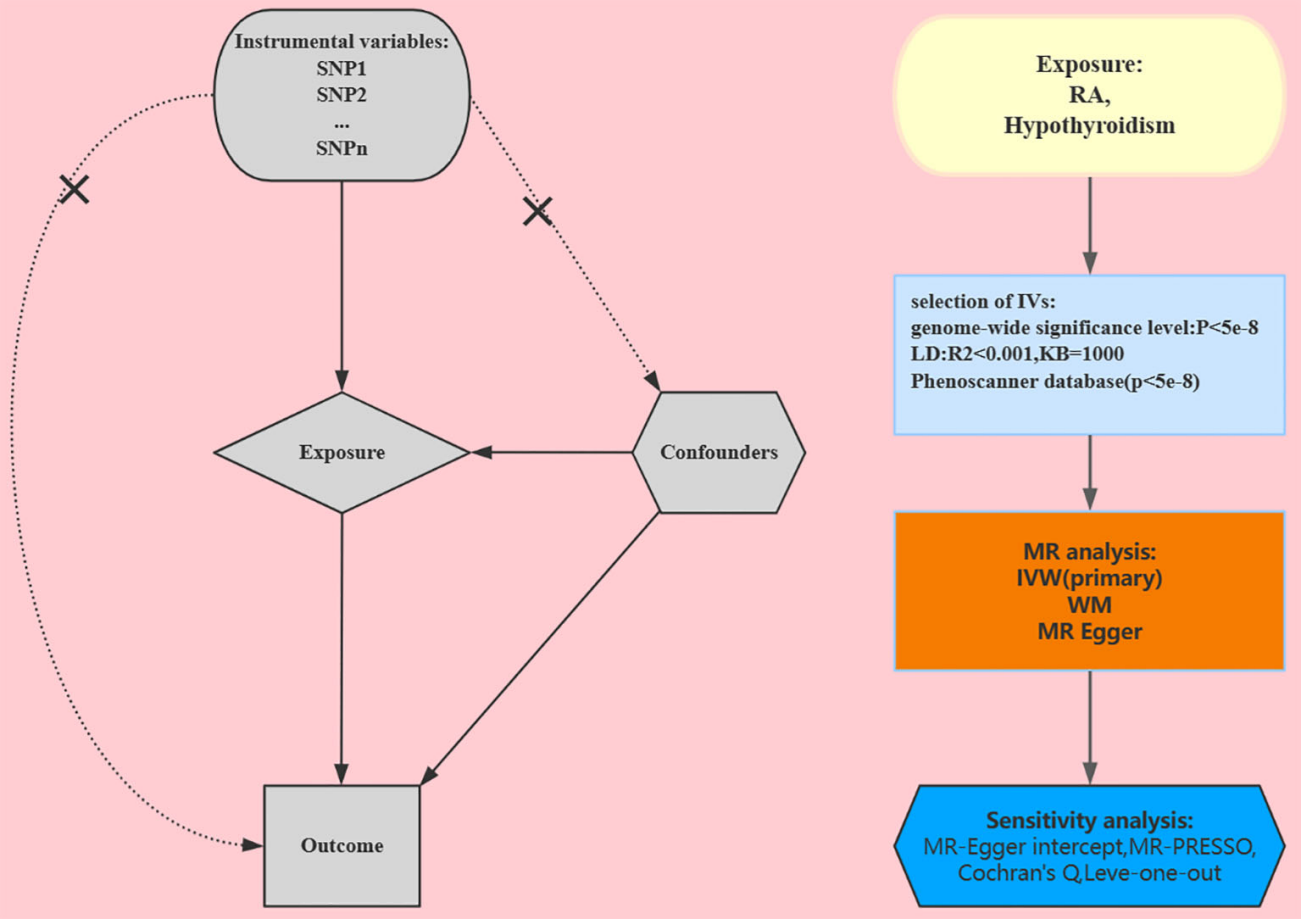
Study design for our Mendelian randomization. RA, rheumatoid arthritis; IVs, instrumental variables; SNP, single-nucleotide polymorphism; WM, weighted median; IVW, inverse variance weighted.

In the European population, to examine the association between RA and hypothyroidism, we used a two-sample MR study. During the MR analysis process, the effective instrumental variables (IVs) must satisfy three key assumptions in order to gain reliable results: 1) The IVs and exposure are tightly associated; 2) The IVs and any confounding factors that might impact both the exposure and the outcome are unrelated; and 3) The IVs only affect the outcome through the exposure (7). To validate each implicational direction, the study involved the following key steps: Genetic IVs associated with appropriate exposures were selected using multiple MR methods, pleiotropy assessment, and heterogeneity and sensitivity analyses. The most recent guidelines (STROBE-MR) (8) were adhered to in this study. **Figure 1** shows a design for a bidirectional MR.

### Data sources

#### GWAS data for RA

To prevent the effects of population stratification, each SNP and its accompanying summary data were extracted from studies that covered only European ancestry. Firstly, summary data for RA (9) from a massive GWAS meta-analysis encompassing 58,284 individuals with European descent (14,361 cases and 43,923 controls) is obtained from the IEU GWAS database (https://gwas.mrcieu.ac.uk/datasets/). All RA cases were either determined to have RA by a qualified rheumatologist or diagnosed with RA according to the 1987 American College of Rheumatology criteria. In addition, we selected another recently published RA dataset from the GWAS Catalog (https://www.ebi.ac.uk/gwas/) to validate our conclusions. It contained 35,871 RA patients and 240,149 control individuals of European, East Asian, African, South Asian, and Arabian ancestry from 37 cohorts; we selected only the European population (25 cohorts: 22,350 cases and 74,823 controls) as our data for analysis (10).

#### GWAS data for hypothyroidism

In the main analysis, from the GWAS ATLAS (https://atlas.ctglab.nl/traitDB), we downloaded summary statistics for hypothyroidism (phenotype field: 20002_1226) and secondary hypothyroidism (phenotype field: 41204_E03), which contained 289,307 and 244,890 individuals from the UK Biobank, respectively (11). In addition, we used summary-level TSH and FT4 data from the Thyroid Omics Consortium because hypothyroidism and circulating TSH and FT4 levels are tightly related (12).

For replication purposes, we also utilized summaries of data from the FinnGen Consortium (https://r7.finngen.fi/) (13). In the FinnGen database (release R7, 2022), we selected hypothyroidism (congenital or acquired), hypothyroidism (levothyroxine purchases), and hypothyroidism (strict autoimmune). There are a total of 309,154 (173,746 females and 135,408 males) participants and 3,095 diseases in the database for the European population. The FinnGen project gives demographic information about the participants, such as their age and gender. The data set is available whenever it is needed. Details on the GWAS data sources used in our study are provided in **Table 1**.

**Table 1:** The GWAS data source details in our study.

Phenotype	Data source	PMID	case	control	Sample size	Ancestry
Hypothyroidism (congenital or acquired)	FinnGen	–	38,624	76,464	115,088	European
Hypothyroidism, strict autoimmune	FinnGen	–	33,422	227,415	260,837	European
Hypothyroidism, levothyroxin purchases	FinnGen	–	38,127	76,464	114,591	European
Rheumatoid arthritis	Okada Y et al.	24390342	14,361	43,923	58,284	European
Rheumatoid arthritis	Ishigaki K et al.	36333501	22,350	74,823	97,173	European
TSH	ThyroidOmics Consortium	30367059	–	–	54,288	European
FT4	ThyroidOmics Consortium	30367059	–	–	49,269	European
Hypothyroidism	ATLAS	31427789	18,740	270,567	289,307	European
Secondary hypothyroidism	ATLAS	31427789	13,043	231,847	244,890	European

FT4, free T4; TSH, thyroid-stimulating hormone.

### Instrumental variable selection

As a first step toward verifying the first hypothesis, genome-wide significant (p <5×10^-8^) SNPs were employed in this study as IVs to evaluate the causal association between RA and hypothyroidism. Second, to remove SNPs linked to significant linkage disequilibrium (LD), we employed a clumping approach with R2<0.001 and a window size of 10 Mb. We then manually searched the PhenoScanner database (http://www.phenoscanner.medschl.cam.ac.uk/phenoscanner) to exclude SNPs that potentially alter the result features. SNPs that were palindromes or ambiguous were eliminated. Additionally, our results were also adjusted for horizontal pleiotropy by applying the MR-Pleiotropy Residual Sum and Outlier (MR-PRESSO) method. We evaluated the strength of the IVs for the screened SNPs using F-statistics and variance (R2) in order to avoid weak-tool bias (F>10): F = R^2^*(N-K-1)/K*(1-R^2^) (14).

### Mendelian randomization analysis

The MR-Egger (15), weighted median (WM) (16), and inverse variance weighted (IVW) (17) techniques were employed in this study to determine the cause-and-effect relationship between RA and hypothyroidism, with the IVW technique predominating. The IVW method is our primary statistical analysis technique, although it could be skewed if IVs display horizontal pleiotropy. Due to this, to examine the reliability of our findings and potential pleiotropy, we conducted sensitivity analyses using the weighted median and MR-Egger regression approaches. The weighted median technique produces unbiased causal estimation if more than 50% of the SNPs are invalid genetic instruments. The MR-Egger regression method is able to provide estimates that have pleiotropy corrections applied to them.

### Sensitivity analysis

Firstly, we employed the MR-Egger intercept to determine whether pleiotropy was present. The heterogeneity of IVW estimates was quantified using Cochran’s Q test. Also, the MR-PRESSO test was run to check for outliers. If outliers are discovered, we will eliminate them and reevaluate the MR effect. In order to evaluate the robustness of the results, we apply the leave-one-out method to remove certain SNPs that have a negative impact on the research outcomes and then recalculate the results. The whole analysis was carried out in RStudio (version 4.2.1) with the “TwoSampleMR” (version 0.5.6) and “MRPRESSO” (version 1.0) packages.

## Results

### Effects of rheumatoid arthritis on hypothyroidism

**Figure 2:**
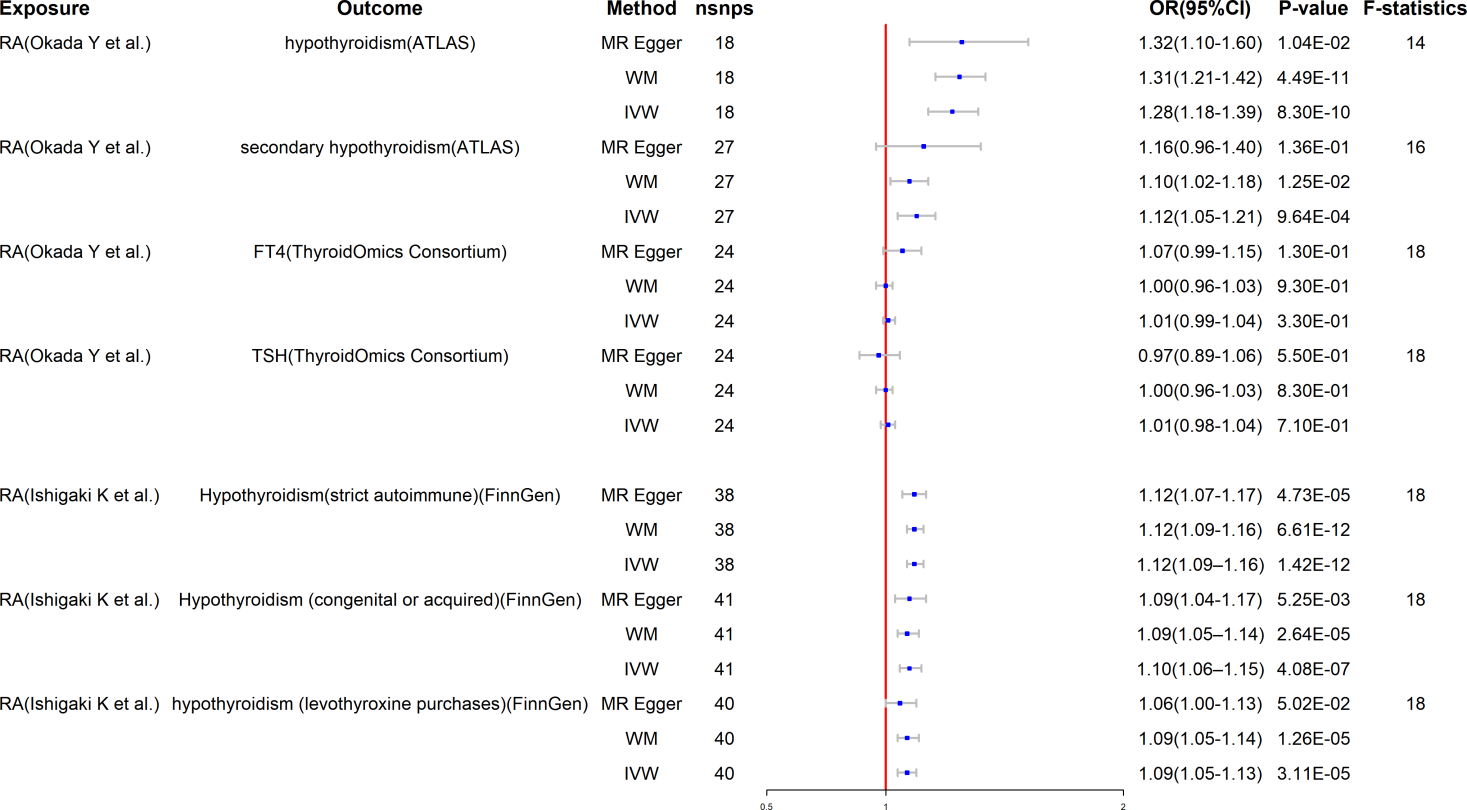
Mendelian randomization for the association of rheumatoid arthritis on hypothyroidism. RA, rheumatoid arthritis; FT4, free T4; TSH, thyroid-stimulating hormone; WM, weighted median; IVW, inverse variance weighted; nSNPs, number of SNPs used in MR; OR, odds ratio; CI, confidence interval.

The MR results of RA on hypothyroidism are listed in **Figure 2**. After using strict criteria to rule out SNPs, we used 18 SNPs for hypothyroidism and 27 SNPs for secondary hypothyroidism. All of these IVs’ F-statistics were greater than 10, indicating that there was no weak instrument bias.

As a result of significant heterogeneity (the p-value for Cochran’s Q is less than 0.05) (**Table 2**), we applied the IVW method to a multiplicative random-effects model. This study demonstrates that genetically predicted RA is significantly related to an elevated risk of hypothyroidism in the main analysis. Specifically, our study demonstrates a substantial relationship between elevated RA and the genetic risk of hypothyroidism (OR = 1.28, 95% CI: 1.18–1.38, P = 8.30E-10) as well as secondary hypothyroidism (OR = 1.12, 95% CI: 1.05–1.21, P = 9.63E-4). Similar results were obtained by using data from the FinnGen Consortium: hypothyroidism (strict autoimmune) (OR = 1.12, 95% CI: 1.09–1.16, P = 1.99E-6), hypothyroidism (congenital or acquired) (OR = 1.10, 95% CI: 1.05–1.14, P = 3.52E-6), and hypothyroidism (levothyroxine purchases) (OR = 1.09, 95% CI: 1.05–1.13, P = 3.11E-5). Additionally, when TSH and FT4 were used as the results of the MR analysis, there was no evidence for a direct causal effect of RA on TSH level (OR = 1.01, 95% CI: 0.98–1.03, P = 0.71) or FT4 level (OR = 1.01, 95% CI: 0.99–1.04, P = 0.33).

**Table 2:** Sensitivity analysis of the associations between rheumatoid arthritis and hypothyroidism.

Sensitivity analysis of RA on hypothyroidism							
Exposures	Outcomes	Heterogeneity test				Pleiotropy test	
		IVW		MR-Egger		MR-Egger intercept	
		Q	pval	Q	pval	Intercept	*P*
RA (Okada Y et al.)	Hypothyroidism	49.7	<0.001	49.3	<0.001	-0.005	0.72
RA (Okada Y et al.)	Secondary hypothyroidism	70.8	<0.001	70.5	<0.001	-0.004	0.75
RA (Okada Y et al.)	FT4	27.2	0.25	25.2	0.29	-0.007	0.19
RA (Okada Y et al.)	TSH	38.1	0.02	37.1	0.02	0.004	0.45
RA (Ishigaki K et al.)	Hypothyroidism (strict autoimmune)	75.6	<0.001	75.6	<0.001	0.0004	0.92
RA (Ishigaki K et al.)	Hypothyroidism (congenital or acquired)	95.0	<0.001	95.0	<0.001	0.0003	0.94
RA (Ishigaki K et al.)	Hypothyroidism (levothyroxin purchases)	89.7	<0.001	86.9	<0.001	0.005	0.28
Sensitivity analysis of hypothyroidism on RA							
Hypothyroidism	RA (Okada Y et al.)	101.0	<0.001	95.0	<0.001	0.036	0.12
Secondary hypothyroidism	RA (Okada Y et al.)	20.6	0.06	17.6	0.04	0.0446	0.22
FT4	RA (Okada Y et al.)	20.2	0.13	19.4	0.11	0.010	0.53
TSH	RA (Okada Y et al.)	42.2	0.04	41.6	0.04	-0.007	0.55
Hypothyroidism (strict autoimmune)	RA (Ishigaki K et al.)	108.9	<0.001	108.7	<0.001	0.004	0.76
Hypothyroidism (congenital or acquired)	RA (Ishigaki K et al.)	46.8	0.009	47.3	0.007	-0.007	0.59
Hypothyroidism (levothyroxin purchases)	RA (Ishigaki K et al.)	30.9	0.16	30.9	0.13	-0.0008	0.96

RA, rheumatoid arthritis; FT4, free T4; TSH, thyroid-stimulating hormone; WM, weighted median; IVW, inverse variance weighted.

### Effects of hypothyroidism on rheumatoid arthritis

**Figure 3:**
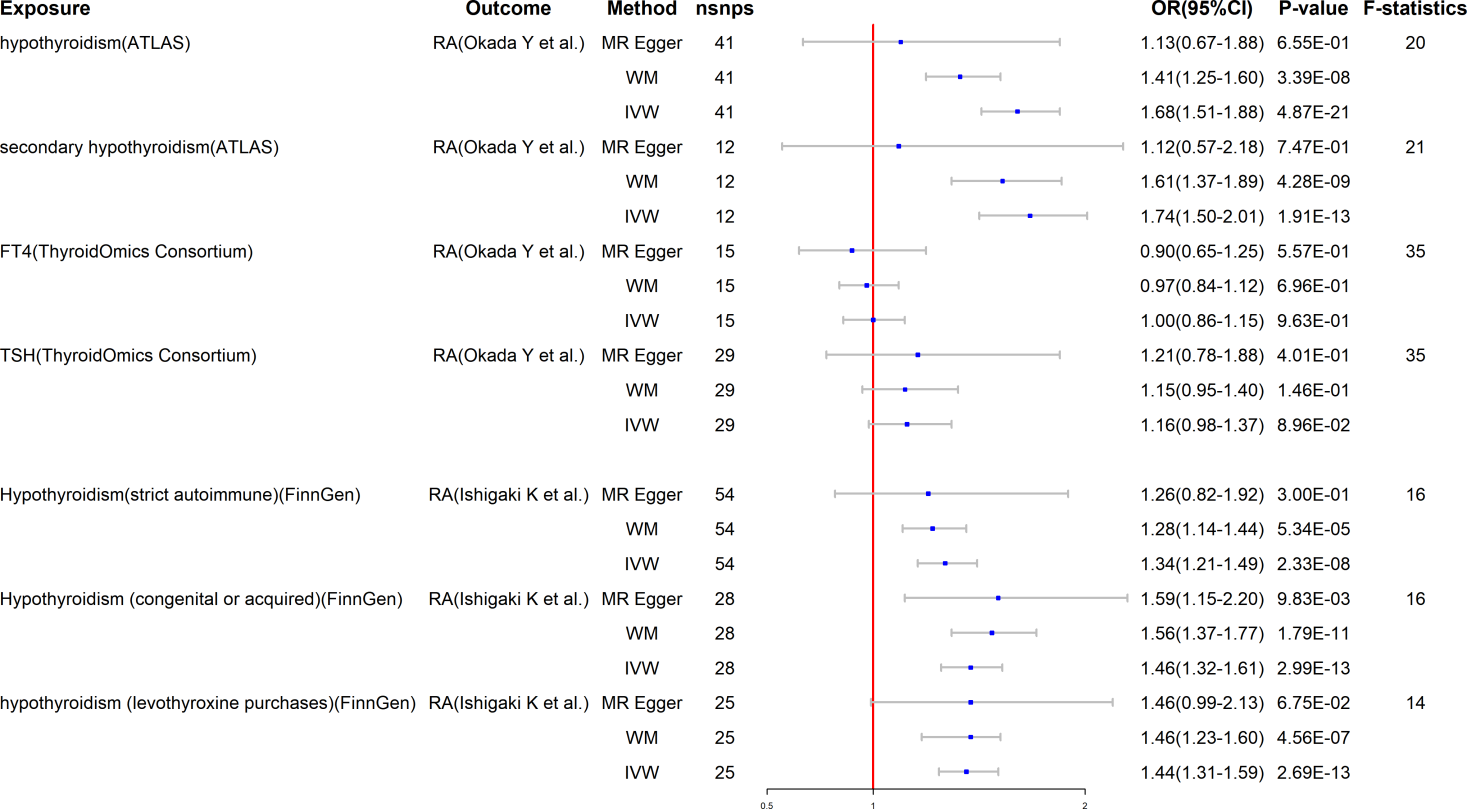
Mendelian randomization for the association of hypothyroidism on RA. RA, rheumatoid arthritis; FT4, free T4; TSH, thyroid-stimulating hormone; WM, weighted median; IVW, inverse variance weighted; nSNPs, number of SNPs used in MR; OR, odds ratio; CI, confidence interval.

Surprisingly, the reverse study yields extremely meaningful results as well. The MR results are listed in **Figure 3**. Hypothyroidism has a strong potential causative relationship with RA, although TSH and FT4 do not have significant causal impacts on RA. Specifically, using the IVW method, the estimated OR for hypothyroidism and secondary hypothyroidism on RA is 1.68 (95% CI: 1.51–1.88, P = 4.87E-21) and 1.74 (95% CI: 1.50–2.01, P = 1.91E-13), respectively. Similar outcomes are obtained with the FinnGen research project. In particular, genetically predicted hypothyroidism (strict autoimmune) (OR = 1.34, 95% CI: 1.21–1.49, P = 2.33E-8), hypothyroidism (congenital or acquired) (OR = 1.45, 95% CI: 1.32–1.61, P = 2.99E-13), and hypothyroidism (levothyroxine purchases) (OR = 1.44, 95% CI: 1.31–1.59, P = 2.69E-13) are strongly correlated with a higher risk of RA. Likewise, neither TSH nor FT4 are causally linked to RA (**Figure 3**). Furthermore, to depict the study data, we employed scatter plots, funnel plots, leave-one-out plots, and forest plots (**Supplementary Materials**).

## Discussion

Consistent with the original study hypothesis, we used MR analysis in this work to find that there is indeed a two-way causal relationship between RA and hypothyroidism. This study used aggregated GWAS data to investigate the causal relationship between RA and hypothyroidism. To our knowledge, this is the first study to estimate the bidirectional causal inference between rheumatoid arthritis and hypothyroidism using magnetic resonance techniques. Compared with previous studies, this study not only found a correlation between RA and hypothyroidism but also provided a reference for the early routine examination of hypothyroidism in future clinical RA patients.

As an autoimmune ailment, RA increases a patient’s risk of developing thyroid issues, notably hypothyroidism (18). 76% of people with RA had one or more extra-articular features, according to a comprehensive investigation. In 6% to 33% of RA patients, thyroid hormone abnormalities and/or autoimmune thyroid illness were detected (19). Hypothyroidism is the most common clinical sign of thyroid hormone deficiency. To diagnose hypothyroidism, the blood TSH and FT4 levels are checked in laboratories. It’s been disputed for a long time (20) whether thyroid dysfunction and rheumatic disease are related (20). Many authors have suggested that hypothyroidism could make rheumatoid arthritis worse (21, 22). The prevalence of thyroid dysfunction was found to be considerably greater in RA patients than controls, according to a case-control research, which is in line with our findings (OR = 2.89, P < 0.001). Additional subgroup analysis found RA and hypothyroidism had a positive connection (OR = 2.28, P = 0.006) (3). Population studies have indicated that autoimmune thyroid illness is more prevalent in RA patients and vice versa (23, 24). An increase in the incidence of hypothyroidism was observed in RA patients, but not an increase in FT4, according to a case-control study. Instead, there is a decline, which is in line with our study’s negative findings regarding FT4 and RA (25). One recent meta-analysis found that RA patients had a threefold higher risk of developing thyroid autoantibodies than healthy controls [thyroglobulin autoantibody (TGAb): OR = 3.17 (2.24-4.49) and thyroperoxidase autoantibody (TPOAb): OR = 2.33 (1.24-4.39)] (26). Using MR Analysis, our study also found a positive correlation between RA and hypothyroidism, and the correlation between the two was significant. In people with RA, thyroid dysfunction has unknown underlying causes (27). It is now widely acknowledged that RA and hypothyroidism can result from a variety of causes, including genetic predisposition and environmental variables. One of the key processes affecting RA and hypothyroidism is genetics. Interestingly, our study found a substantial relationship between elevated RA and genetic risk factors for hypothyroidism in the analysis of genetic variables. According to a study, people with RA are more likely than people with other types of thyroid disease to experience hypothyroidism. A population-based retrospective cohort study that looked into the risk of hypothyroidism in RA patients discovered that the incidence of the condition was 1.74 times higher in the RA group than in controls, with an adjusted hazard ratio of 1.67 (95% CI =1.39-2.00) according to the Cox method after adjusting for covariates. In all categories, the incidence of women was 3.6 times higher than that of men, making up over 75% of the study population (28). The above studies indicate that RA is significantly correlated with hypothyroidism, which is consistent with our findings, but the specific influencing factors and pathogenesis still need to be further studied.

Patients with hypothyroidism frequently experience various rheumatic symptoms, including the possibility of fatigue, myalgia, and joint pain in RA (21, 29). Numerous investigations have discovered that increased disease activity in RA patients and blood tumor necrosis factor-α levels have an effect on hypothyroidism (4, 30). In light of the aforementioned research, it is clear that early thyroid function testing and active therapy have significant clinical implications (31). Additionally, studies have shown a correlation between the disease activity score -28 (DAS-28) and hypothyroidism in RA patients. Hypothyroidism may increase disease activity due to an increase in joint pain, so RA patients should be aware of thyroid function screening for early detection and better treatment (32). One of the most prevalent co-morbidities of RA is hypothyroidism; however, the shared pathophysiology between the two conditions is still unknown, and genes implicated in the control of T cell responses may be important (33). Surprisingly, our study found that hypothyroidism is closely related to the increased risk of RA genetically, which may provide new ideas and directions for our research on the mechanism. In addition, Autoimmunity is a factor in both RA and hypothyroidism. Hypothyroidism is associated with an inflammatory response and pro-inflammatory cytokines such as IL-1 and IL-6 (34, 35). According to studies, there is a relationship between autoimmune diseases and the pathogenesis of RA, as patients with hypothyroidism in their family have a higher risk of developing RA (36). Thyroglobulin antibodies (TgAb) and thyroid peroxidase antibodies (TPOAb) with positive results have been found in RA patients (37). Additionally, inconsistent results have been found in research on the possibility of thyroid malfunction in people with RA (38). To sum up, this study found that RA and hypothyroidism are related to the pathogenesis of genetics, genes and autoimmunity, especially the significant correlation between genetic variables. Although no relationship was found with environmental variables, this study will continue to further explore and study the relationship between the pathogenesis of the two in the future.

The advantage of the bidirectional approach is that it enables the inference of both causal axes between RA and hypothyroidism. The MR is built using the publicly available GWAS summary statistics. Without incurring additional fees for experiments, it is practical for mining reliable genetic data. Second, we validated our findings using extra datasets to make our conclusions more credible. Another benefit is that, for the first time, we utilized MR to find out if RA susceptibility is linked to a higher risk of thyroid disease. The findings of this study indicate that RA is related to an elevated risk of hypothyroidism.

There are also more limitations to our study. Firstly, the samples for RA and hypothyroidism were quite small, which may have caused some false positives and negatives. The causal relationship between RA and hypothyroidism was not stratified by age or gender, which is the second flaw. Thirdly, the exposure and outcome analyses included only Europeans in the study population. It has to be determined if this outcome might be repeated in other populations. Some heterogeneity existed in the study. As a result, the conclusions are less credible because we were unable to reduce the heterogeneity. Other ethnic groups also need to have MR studies conducted, and these studies must be supported by extensive GWAS data.

Finally, RA and hypothyroidism shared a bidirectional causal connection. Our findings suggest that RA patients should routinely be checked for hypothyroidism. At present, there are few studies on RA and hypothyroidism, so it is suggested that future studies focus on the mechanisms between them.

## Data availability statement

The original contributions presented in the study are included in the article/**Supplementary Material**. Further inquiries can be directed to the corresponding authors.

## Ethics statement

Ethical review and approval was not required for the study on human participants in accordance with the local legislation and institutional requirements. Written informed consent from the participants’ legal guardian/next of kin was not required to participate in this study in accordance with the national legislation and the institutional requirements.

## Author contributions

DC, YS: conception, technique, formal analysis, data collection, first draft preparation, and visualization; LD, SY: preparation of the first draft preparation, visualization, and data curation in writing; SD, YF: editing, monitoring, and writing evaluation. The final draft of the manuscript was approved by all writers, who also made contributions to the work.
