# Supplementary material for: Rheumatoid arthritis and hypothyroidism: a bidirectional Mendelian randomization study
Source: Front Immunol. 2023 Aug 2;14:1146261. doi: 10.3389/fimmu.2023.1146261 (PMC10433205; doi:10.3389/fimmu.2023.1146261)
Supplement: Supplementary file 1 [file DataSheet_1.docx]

Supplementary Material

Rheumatoid arthritis and hypothyroidism: A bidirectional Mendelian randomization study


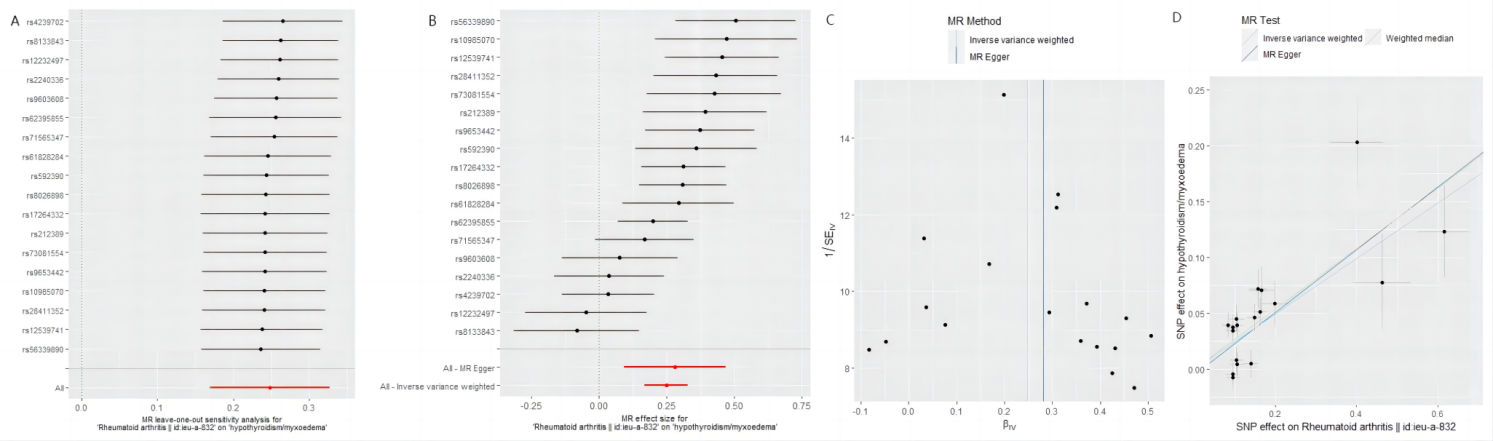


**Supplementary Figure 1.** MR plots for the causal association of RA on hypothyroidism. (A) The leave-one-out sensitivity analysis: each dot and its corresponding line represent the pooled estimates after the removal of corresponding SNP. (B)Forest plot: each dot and its corresponding line represent the effect size and 95%CI. Each dot and its corresponding line represent the pooled estimates after the removal of the corresponding SNP. (C) Funnel plot: the x-axis represents β, and the y-axis represents 1/SE (standard error) . (D) Scatter plots: the estimate of intercept can be interpreted as an estimate of the average pleiotropy of all single-nucleotide polymorphisms (SNPs), and the slope coefficient provides an estimate of the bias of the causal effect.


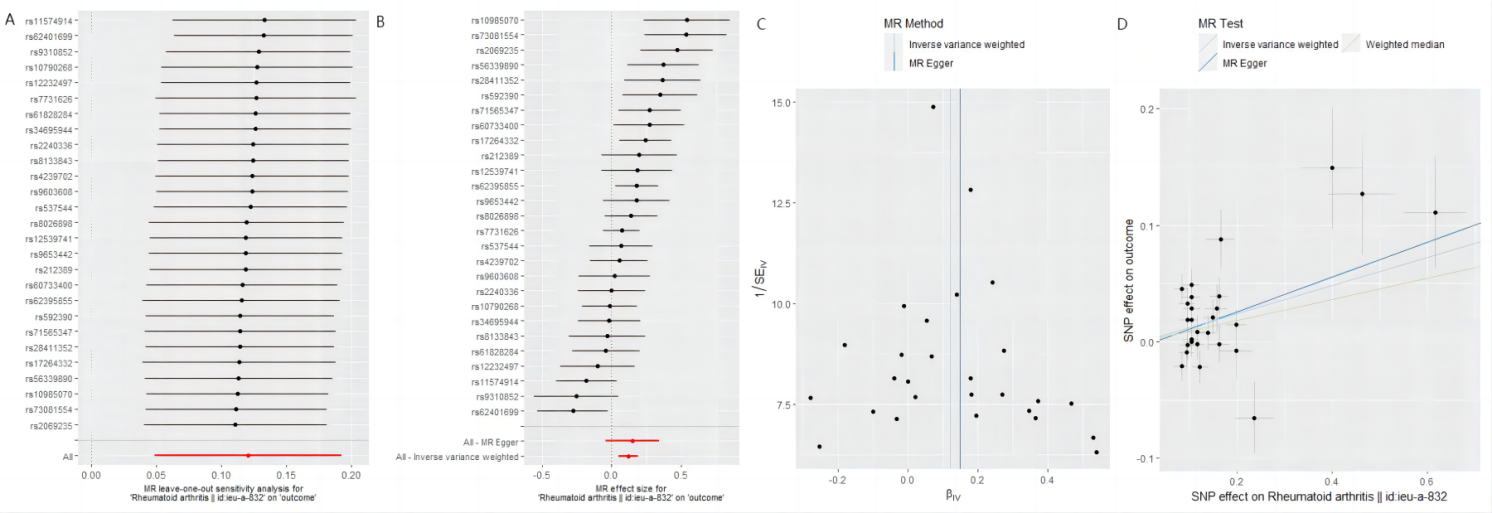


**Supplementary Figure 2.** MR plots for the causal association of RA on secondary hypothyroidism. (A) The leave-one-out sensitivity analysis. (B)Forest plot. (C) Funnel plot. (D) Scatter plot.


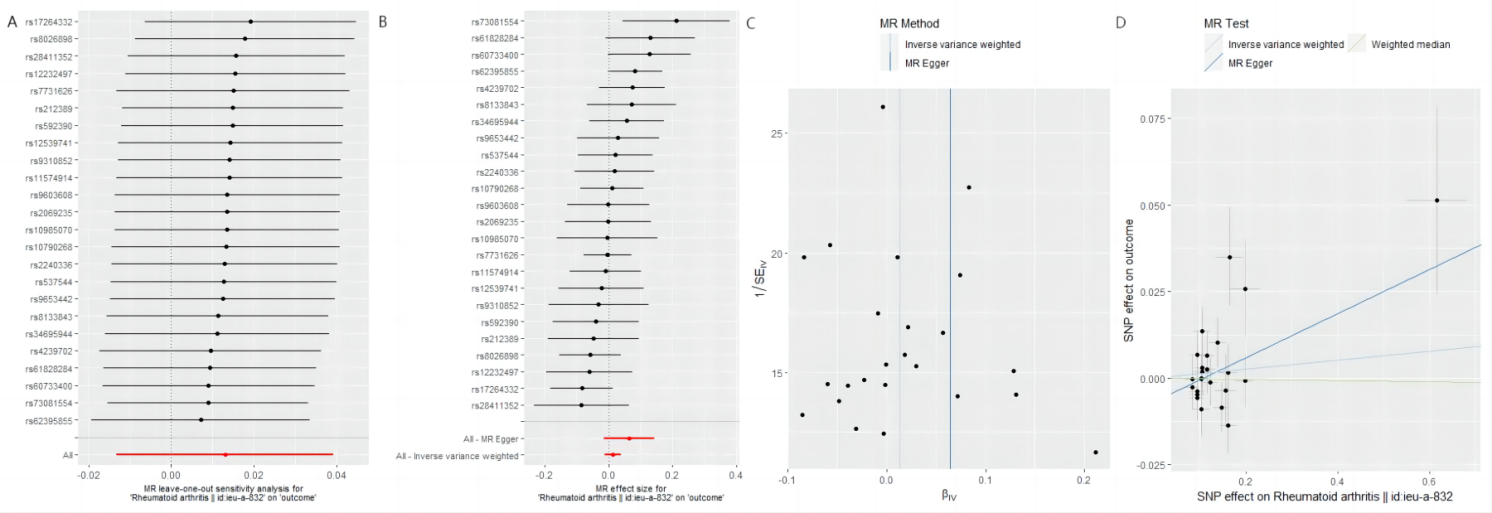
**Supplementary Figure 3.** MR plots for the causal association of RA on FT4.(A) The leave-one-out sensitivity analysis. (B)Forest plot. (C) Funnel plot. (D) Scatter plot.


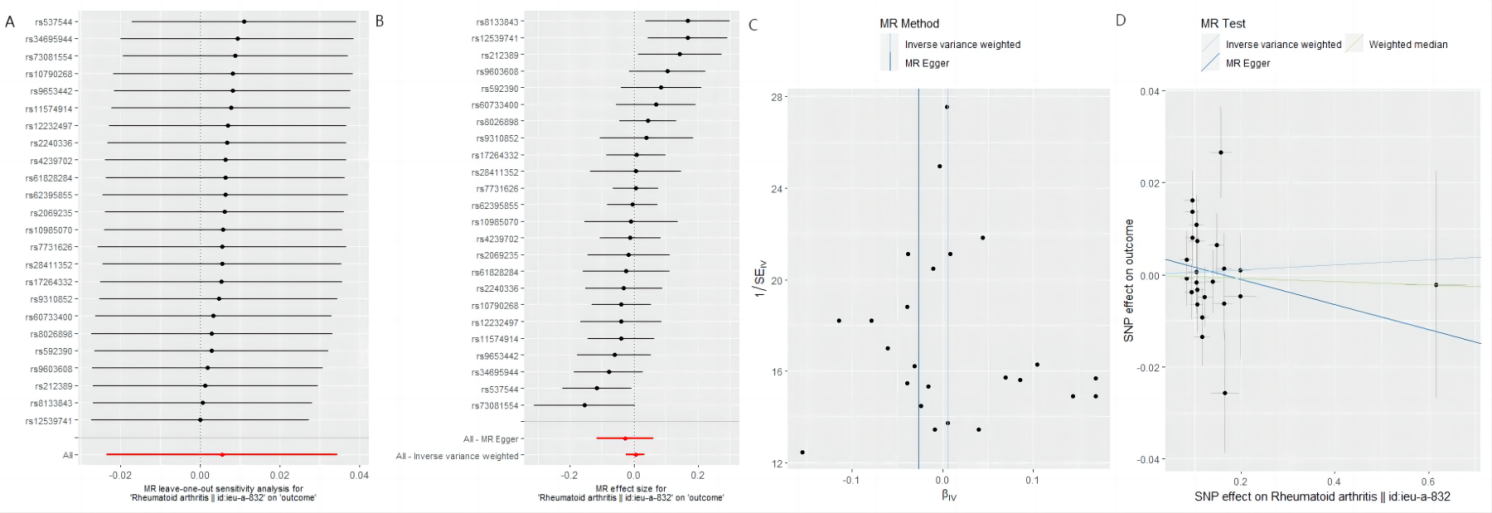


**Supplementary Figure 4.** MR plots for the causal association of RA on TSH. (A) The leave-one-out sensitivity analysis. (B)Forest plot. (C) Funnel plot. (D) Scatter plot.


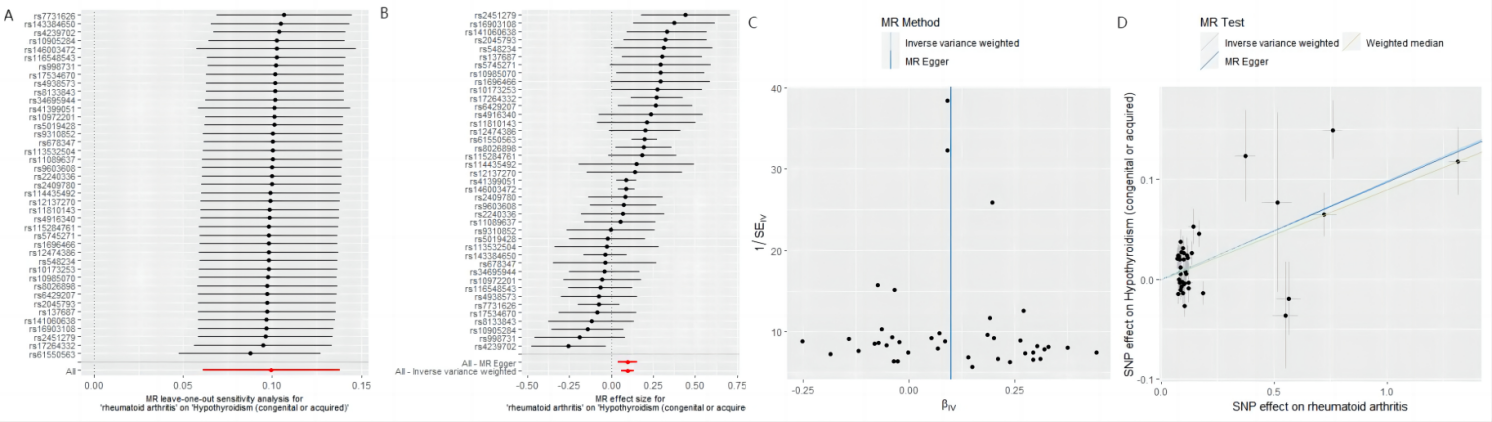
**Supplementary Figure 5.** MR plots for the causal association of RA on hypothyroidism (congenital or acquired) . (A) The leave-one-out sensitivity analysis. (B)Forest plot. (C) Funnel plot. (D) Scatter plot.


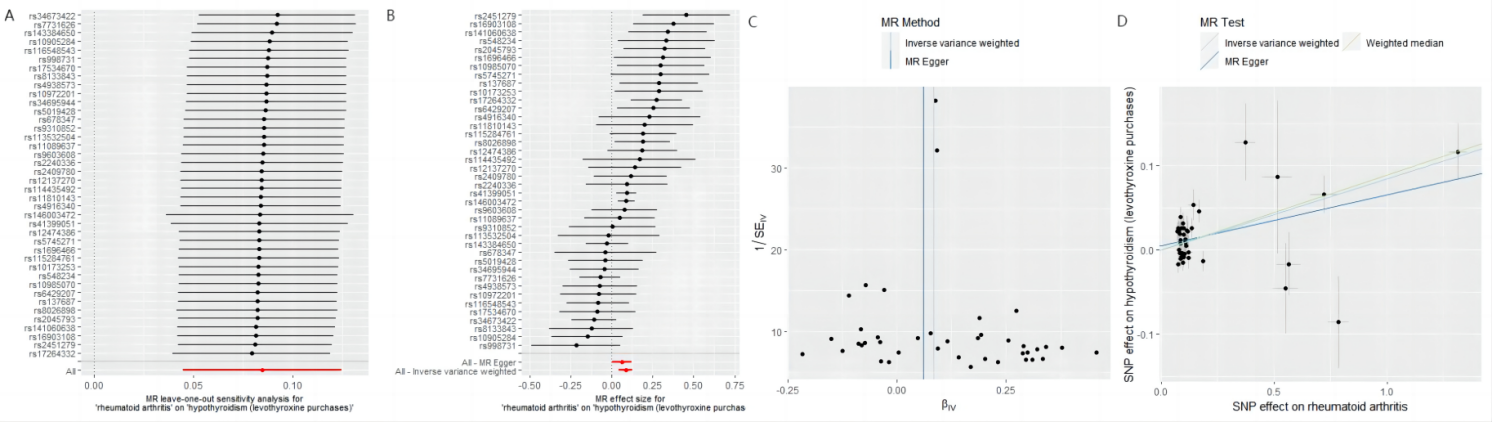
**Supplementary Figure 6.** MR plots for the causal association of RA on hypothyroidism (levothyroxine purchases).(A) The leave-one-out sensitivity analysis. (B)Forest plot. (C) Funnel plot. (D) Scatter plot.


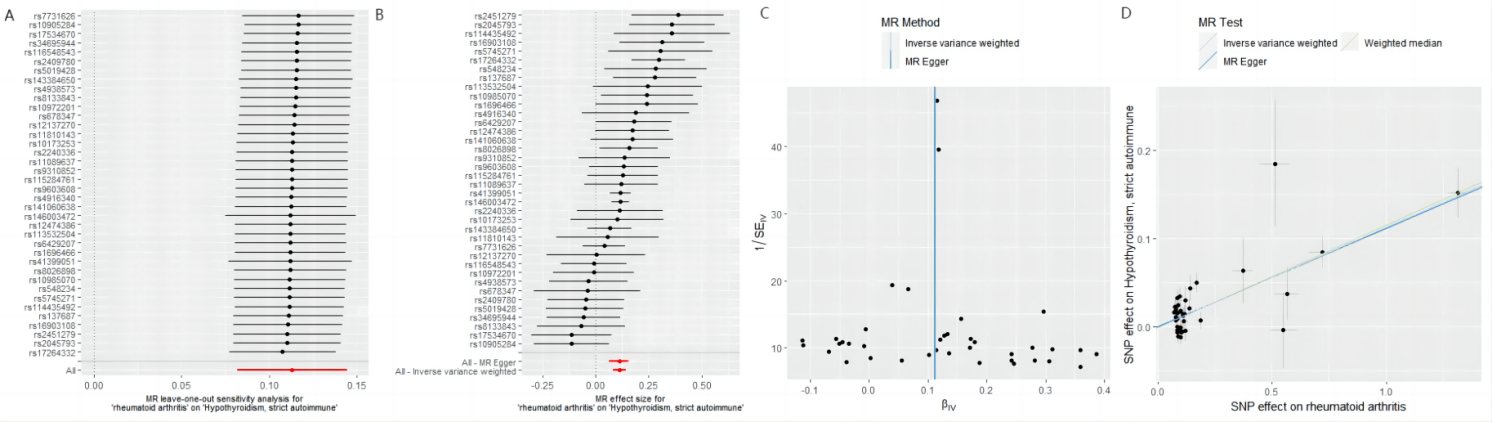
**Supplementary Figure 7.** MR plots for the causal association of RA on hypothyroidism (strict autoimmune).(A) The leave-one-out sensitivity analysis. (B)Forest plot. (C) Funnel plot. (D) Scatter plot.


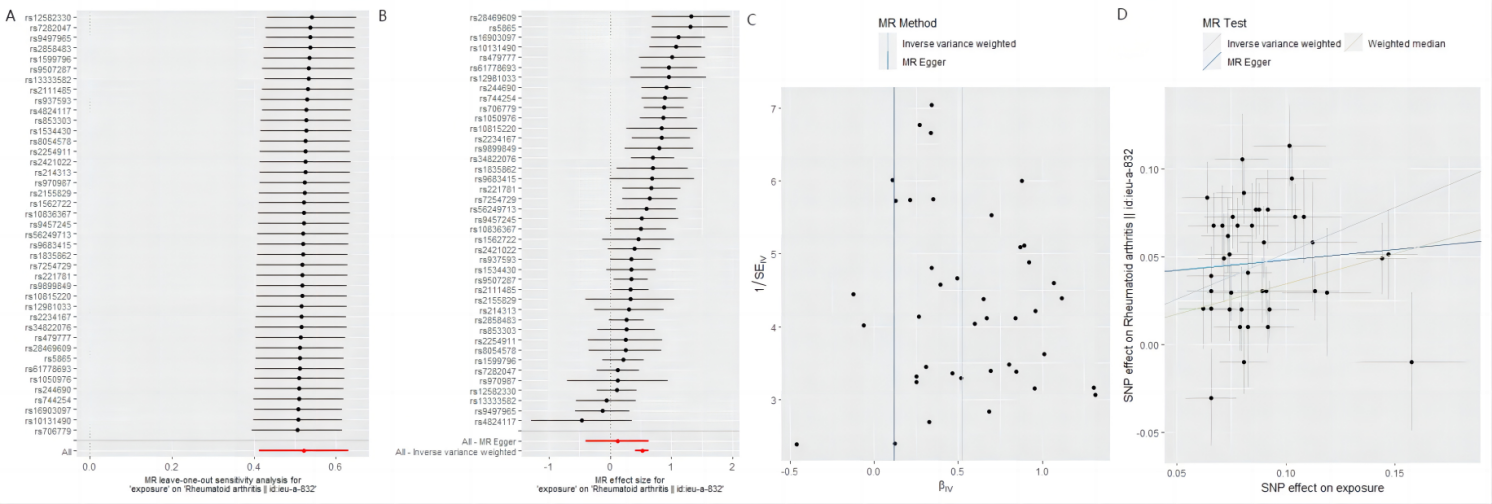


**Supplementary Figure 8.** MR plots for the causal association of hypothyroidism on RA. (A) The leave-one-out sensitivity analysis. (B)Forest plot. (C) Funnel plot. (D) Scatter plot.


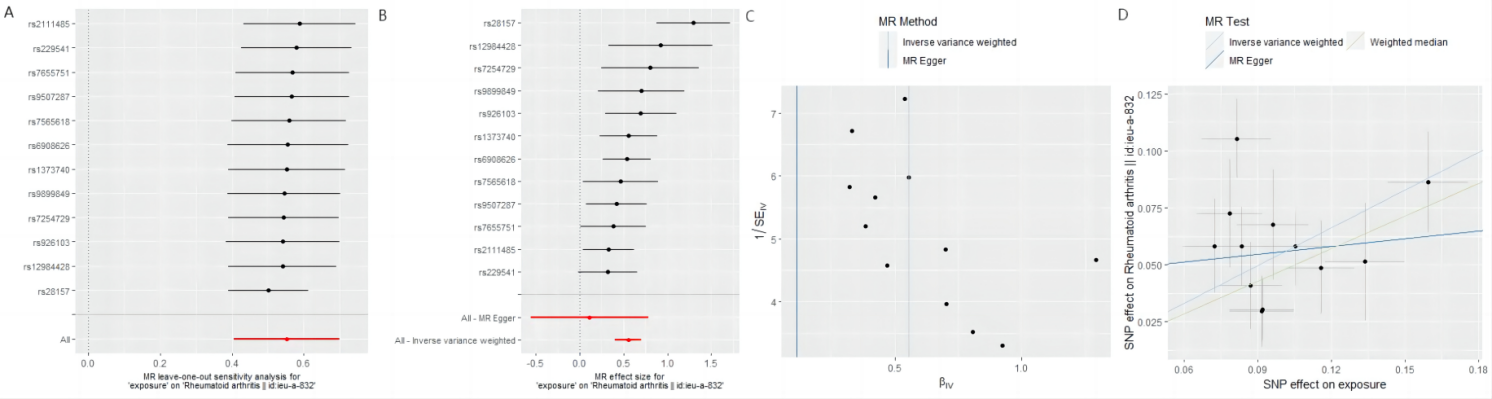


**Supplementary Figure 9.** MR plots for the causal association of secondary hypothyroidism on RA. (A) The leave-one-out sensitivity analysis. (B)Forest plot. (C) Funnel plot. (D) Scatter plot.


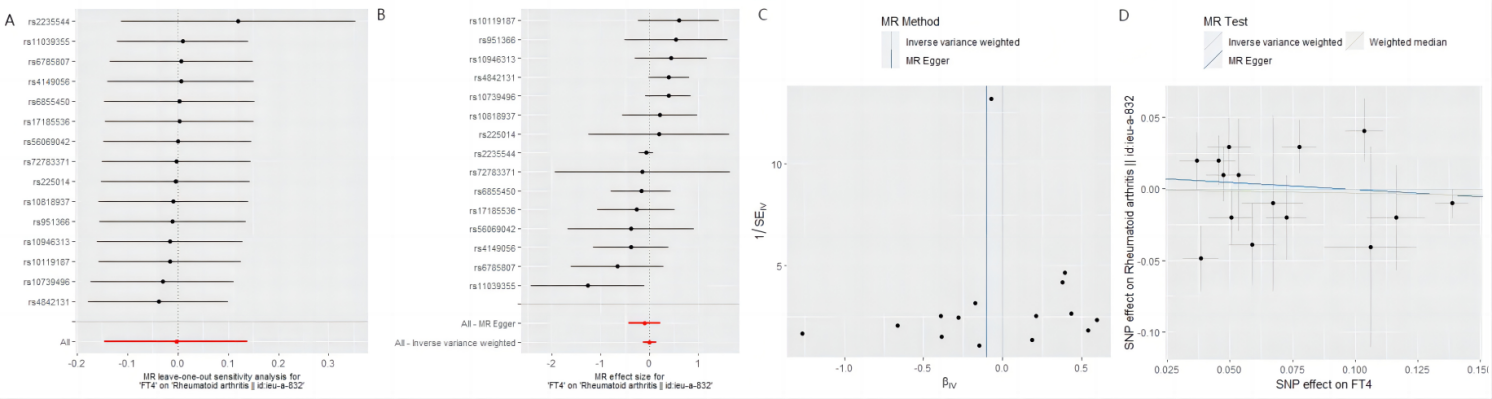


**Supplementary Figure 10.** MR plots for the causal association of FT4 on RA. (A) The leave-one-out sensitivity analysis. (B)Forest plot. (C) Funnel plot. (D) Scatter plot.


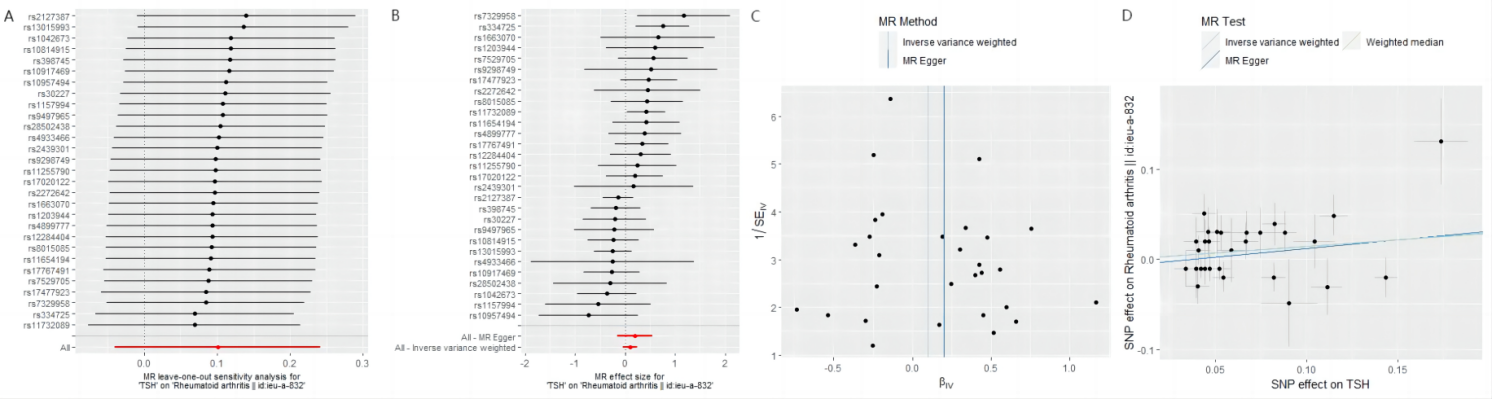


**Supplementary Figure 11.** MR plots for the causal association of TSH on RA. (A) The leave-one-out sensitivity analysis. (B)Forest plot. (C) Funnel plot. (D) Scatter plot.


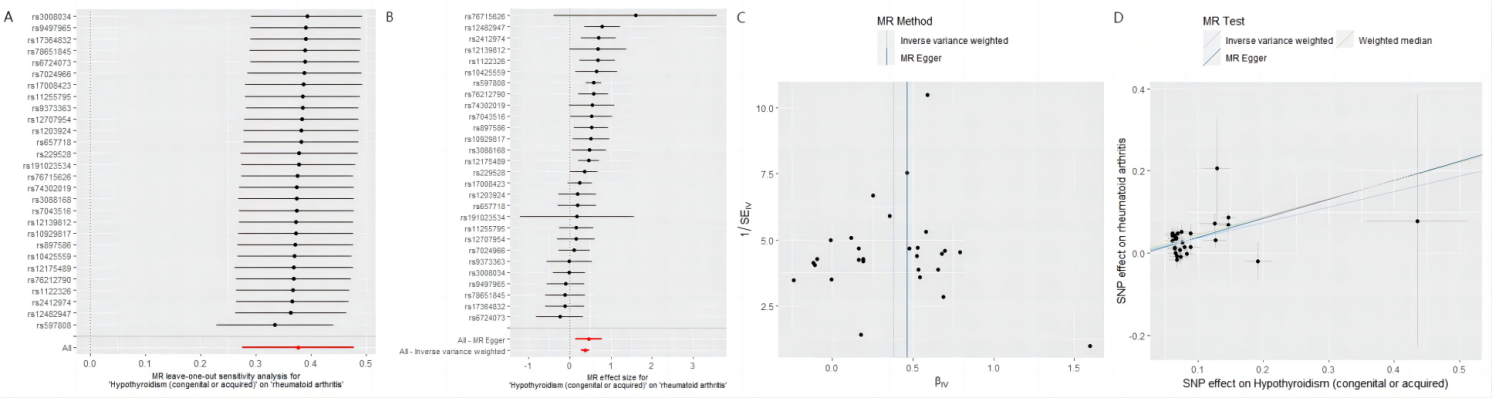


**Supplementary Figure 12.** MR plots for the causal association of hypothyroidism (congenital or acquired) on RA. (A) The leave-one-out sensitivity analysis. (B)Forest plot. (C) Funnel plot. (D) Scatter plot.


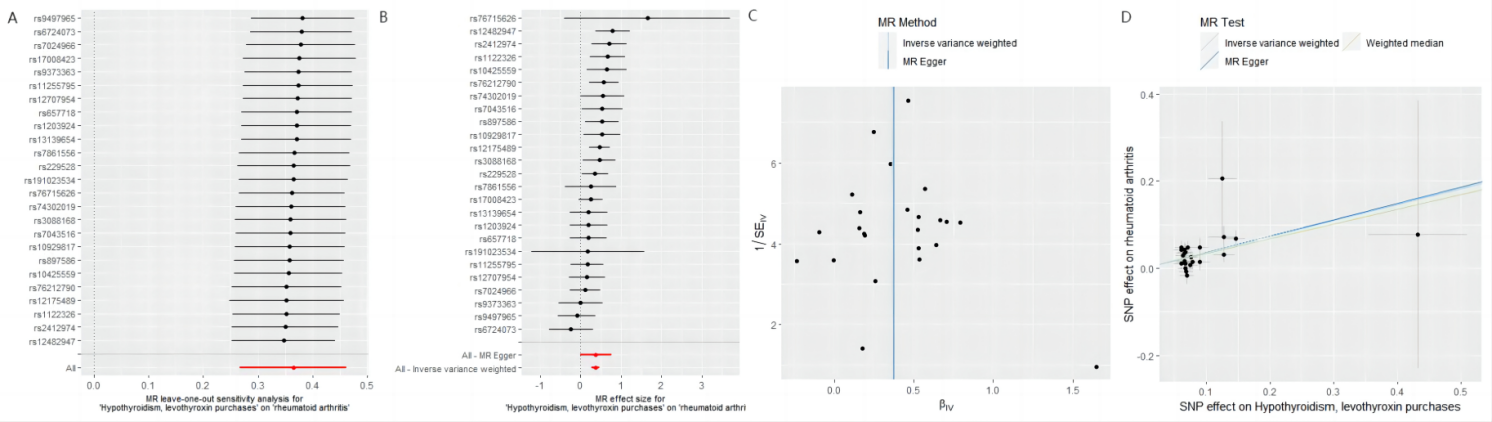


**Supplementary Figure 13.** MR plots for the causal association of hypothyroidism (levothyroxine purchases) on RA. (A) The leave-one-out sensitivity analysis. (B)Forest plot. (C) Funnel plot. (D) Scatter plot.


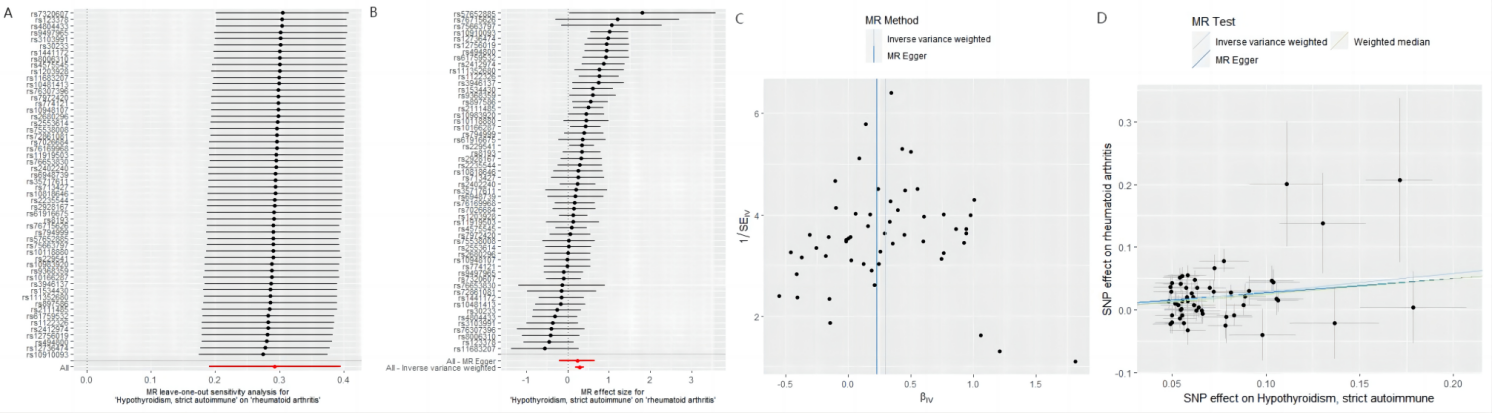


**Supplementary Figure 14.** MR plots for the causal association of hypothyroidism (strict autoimmune) on RA. (A) The leave-one-out sensitivity analysis. (B)Forest plot. (C) Funnel plot. (D) Scatter plot.
